# The Mediating Effect of Childcare Teachers’ Resilience on the Relationship between Social Support in the Workplace and Their Self-Care

**DOI:** 10.3390/ijerph17228513

**Published:** 2020-11-17

**Authors:** Nam-Shim Park, Seung-Min Song, Jung Eun Kim

**Affiliations:** 1 Michael Angel Pre-kindergarten, Suwon-si 16681, Korea; hayoung-sky@hanmail.net; 2 Department of Child and Family Welfare, University of Suwon, Hwaseong-si 18323, Korea; smsong@suwon.ac.kr

**Keywords:** self-care, resilience, social support, childcare teachers, mediation analysis

## Abstract

(1) Background: The purpose of this study is to examine the relationship between social support in the workplace for childcare teachers, resilience, and self-care. This study explores the inner mechanism that helps to strengthen self-care of childcare teachers, which enables teachers to provide quality care to children and promote their own wellbeing. (2) Methods: The survey was conducted from September to October 2018 for childcare teachers in Seoul and Gyeonggi Province using convenience sampling. Out of 550 questionnaires, 491 were returned, with 466 used for the analysis, excluding those with incomplete responses. The collected data were analyzed using descriptive statistics, correlation analysis, and mediation analysis. (3) Results: There were significant correlations between all variables. The mediation analysis showed a complete mediation of resilience. (4) Conclusion: Childcare teachers first have to take good care of themselves in order to perform well as a childcare professional. Educational materials and counseling programs tailored for childcare teachers need to be developed for better self-care and building greater resilience. Materials for directors of daycare centers, as well as teachers stressing the importance of social support for each other, will help childcare teachers’ effective functioning in their professional and personal life. Prevention and intervention programs for self-care will eventually help lower the costs of healthcare in society.

## 1. Introduction

Self-care is the personal ability to maintain psychological and emotional balance. It builds one’s holistic health and wellbeing, thus contributing to taking care of others [1,2,3]. Self-care is necessary for a caregiver to provide better care to others and self-care behavior is known to be related to professional, as well as personal, success [4]. Failure of self-care by professionals in caregiving jobs may result in them putting themselves or their clients in danger [2]. Promoting self-care can help caregivers cope with work-related stress and enable them to achieve both professional development and success, not to mention, personal wellbeing [4]. Self-care both prevents and protects against negative outcomes in the personal and professional domains, which helps to lessen the costs of healthcare from a societal perspective. This emphasizes the importance of the health and wellbeing of the entire population [5].

Caregivers such as childcare teachers and those who provide mental/physical health services are consistently confronted with a high risk of negative consequences such as psychological distress [6,7]. In the process of helping others to have positive outcomes, caregivers tend to disregard their own needs [2]. Professionals in this field must make caring for and nourishing themselves a priority in order to function effectively and provide optimal care for clients [8,9]. Studies in mental and physical health care services focusing on counselors and therapists [2,4,10,11], as well as on nurses [12,13], have already noticed the importance of self-care, although little attention has been paid to childcare teachers who also provide primary care for young children and have a critical role in child development.

Due to the recent increase in the number of employed mothers, young children in South Korea stay at daycare centers for an average of 7–11 hours [14], while approximately 86% of children younger than age 5 attend childcare centers in Seoul and the metropolitan area [15]. In this light, it is no exaggeration to say that childcare teachers are the primary caregivers of young children as they spend nearly a third of the day with young children. Hence, the psychological and emotional states of childcare teachers have a significant influence on children, particularly considering the rapid and critical change in social and emotional development during this period [16], as childcare teachers’ mental stability provides a sense of stability for young children.

Self-care allows childcare teachers to recognize their personal and professional identity, maintain a psychological balance, and accept children as they are [17] since self-care enables teachers to function effectively thanks to being equipped with the qualities of good teachers such as enthusiasm, creativity, flexibility, patience, ability to respect differences in personality and learning style, and so forth [18]. Considering the importance of early childhood education, the psychological stability of a childcare teacher increases young children’s emotional development and stable attachment to form a positive self-concept [19]. However, childcare teachers in Korea said it was difficult to find time to look after themselves due to heavy workloads [20]. The number of young children per childcare teacher was reported to be 6.3 in Korea, while the ratio of child-to-teaching staff for young children aged 3–5 is higher than in other OECD countries [21]. Not surprisingly, work overload was ranked one of the top categories among sources of job stress among childcare teachers in Korea [22,23].

Research has stressed the importance of self-care since mental health problems, such as depression, anxiety, and stress, arise when a counselor’s self-care is insufficient [24]. Mental health problems cause instability in psychological and emotional states and problems in interpersonal relationships [25,26,27]. Hence, childcare teachers with poor mental health may become passive in their jobs and less sensitive while interacting with young children, both of which can have a detrimental impact on the child’s development [28,29]. For instance, teachers with depression or anxiety often abruptly finished play so that such activities cannot be linked or expanded to other activities with children [27,30,31]. In addition, mental health problems resulting from poor self-care are very likely to lead to an increase in physical symptoms such as insomnia, fatigue, and headaches [32,33]. These symptoms affect the counselor’s job performance, which subsequently results in negative outcomes for clients’ wellbeing [34]. The scenario is likely to be the same for childcare teachers; there is a high probability that the negative emotions of teachers are transmitted to young children and that teachers may not effectively and actively participate in caregiving work.

As daycare centers are generally located in smaller spaces, close cooperation with fellow teachers and directors is important as it increases the psychological stability and positive self-awareness of childcare teachers [35,36,37]. Social support in the workplace includes assistance and resources that can be obtained from colleagues and supervisors surrounding an individual [38,39], while social support in the workplace of childcare teachers was reported to prevent burnout [36,40]. When teachers believed that they had received a fair evaluation from their colleagues and supervisors and when they received love, respect, and interest from fellow teachers and directors, they showed higher levels of self-confidence [41]. Other studies regarding patients or medical professionals such as nurses found that social support lessened the probability of neglecting self-care [42,43]. However, there is little research on the relationship between the self-care of childcare teachers and the social support they receive in the workplace. Thus, this study examined the relationship between the two variables, both of which are considered essential for the quality development of children.

It has been reported that self-care is associated with psychological factors [44,45,46,47]. In particular, resilience allows for maintaining psychological balance and preserving psychological wellbeing, which is related to effective self-care [3,48]. Resilience is the ability to adapt well and overcome adversity or stressful and challenging situations [49,50,51]. It is an essential skill for childcare teachers since they are more stressed than other occupations due to role conflict, employment insecurity, and low awareness [52]. Studies have found that teachers with low resilience showed a significantly lower level of self-confidence, hope for the future, and confidence in their work [44]. Studies using a sample of nurses showed similar results; respondents’ characteristics such as hope, self-efficacy, coping, and competence were significantly related to the level of resilience [48,53]. Higher resilience was related to the ability to bounce back from disadvantaged circumstances and showed a positive effect on trauma recovery [48]. Accordingly, low resilience has a negative impact on psychological, emotional, and physical health [54,55]. Low resilience may lead to the loss of a sense of balance in life and thereby result in a lack of control or managing skills, which can be interpreted as a failure in self-care. Studies examining the relationship between resilience and self-care for childcare teachers are limited, although a study on helping nurses maintain their level of resilience found that the level of self-care got better as the level of resilience improved [56]. In the case of primary caregivers for the elderly, self-care and satisfaction with themselves improved as resilience increased [57].

Resilience of teachers is greatly affected by social resources in the workplace provided by directors and colleagues [58]. In regard to daycare centers, the influence of social support would be greater since teachers interact in a relatively limited space for more than 9 h per day. Social support resources in the workplace act as an inner protective factor for childcare teachers to respond wisely to negative situations and to perceive life more positively [59,60,61]. A positive correlation was reported between social support and resilience [62,63]. In addition, social support in the workplace showed a direct effect on resilience. The intimacy and attention obtained from supportive resources in the workplace had a positive effect by improving resilience [35,64]. A closer review of previous studies reveals that the advice and praise of directors and fellow teachers helped childcare teachers to improve their resilience, control their own emotions, understand others’ emotions, and make them feel confident about themselves in regard to their professional and personal life [59].

**Figure 1:**
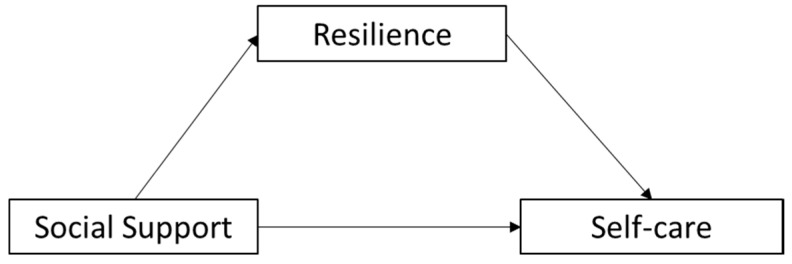
Hypothesized Model.

The relationships between self-care, social support in the workplace, and resilience have been reviewed in this section, while research examining the relationships among the three has not been conducted. Moreover, studies focusing on self-care for childcare teachers have been scarce, despite it being one of the most stressful occupations [52]. It has been reported that it was hard for them to find time to care and nourish themselves [20], which results in them neglecting their own mental and physical health. The current study focused on childcare teachers and investigated the mediating role of resilience in the path between social support and self-care. Based on the review of the literature, it was found that (a) social support in the workplace and resilience had influences on self-care and (b) that social support in the workplace affected resilience. Therefore, it is expected that social support has an indirect effect on self-care through resilience. Research hypotheses and the hypothesized model (Figure 1) are as follows.

**Hypothesis** **1.** *Childcare teachers’ self-care, resilience, and social support in the workplace will be significantly and positively correlated with each other.*

**Hypothesis** **2.** *Resilience of childcare teachers will mediate the relationship between social support in the workplace and self-care.*

**Hypothesis** **2a.** *Social support in the workplace will show a significant and direct effect on the resilience of childcare teachers.*

**Hypothesis** **2b.** *The resilience of childcare teachers will show a significant effect on self-care.*

## 2. Materials and Methods

### 2.1. Data Collection and Subjects

This study targeted 466 childcare teachers currently working at daycare centers in Seoul and Gyeonggi Province, South Korea. The survey was conducted from 24 September to 19 October 2018, using convenience sampling after the pilot survey was conducted in early August (IRB File No. 1808-045-02). Researchers visited or called the directors of daycare centers in Seoul and Gyeonggi Province to explain the purpose of the research and recruit participants. With the approval of the directors, the questionnaire was distributed to childcare teachers along with a survey consent form and a gift (valued at approximately US$5) was provided as compensation for participants. A total of 550 questionnaires were distributed to childcare teachers, with 491 questionnaires returned (89.3% response rate). Questionnaires with incomplete responses were excluded and a total of 466 were used for the analysis.

The demographic characteristics of the subjects are as follows. Of the 446 subjects, 259 teachers (55.6%) were in their 40s or older, 214 (45.9%) had graduated from a vocational college, 209 (44.8%) had less than 5 years of work experience, and 261 (56.0%) had a monthly income of less than 2 million Korean won (KRW), approximately US$1,800. The average amount of monthly income was 2.3 million KRW, while 331 (71.0%) worked 9 h per day and 375 (80.5%) were married. Details of the characteristics of subjects are presented in Table 1.

### 2.2. Measures

#### 2.2.1. Self-Care

Health Promoting Life Profile II (HPLPII) developed by Walker, Sechrist, and Pender [65,66] was used to measure the level of self-care of childcare teachers. Since a self-care scale developed for childcare teachers was not available, we selected HPLPII, which is one of the most commonly used measures in studies of health [67]. HPLPII generated items to assess self-initiated activities and perceptions that can help maintain and enhance individuals’ wellness [65,66]. We used the Korean version of HPLPII as translated by Kim [68] based on the previous version of Yun and Kim [69].

The scale comprised of 52 questions in 6 categories regarding health promoting behaviors: health responsibility (9 questions), physical activity (8 questions), nutrition (9 questions), spiritual growth (9 questions), interpersonal relations (9 questions), and stress management (8 questions). Each area included the following statement: “Report any unusual signs or symptoms to a physician or other health professional” for health responsibility; “Take part in light to moderate physical activity (such as sustained walking 30–40 min 5 or more times a week” for physical activity, “Eat 2~4 servings of fruit each day” for nutritional intake, “Feel I am growing and changing in positive ways” for spiritual growth; “Discuss my problems and concerns with people close to me” for interpersonal relation efficiency; and “Use specific methods to control my stress” for stress management. Each question was measured with a 4-point Likert scale (1 = “never,” 2 = “sometimes,” 3 = “often,” and 4 = “routinely”). Cronbach’s α coefficient was 0.94 for self-care, 0.82 for health responsibility, 0.90 for physical activity, 0.75 for nutrition, 0.82 for spiritual growth, 0.82 for interpersonal relations, and 0.77 for stress management, respectively.

#### 2.2.2. Social Support in the Workplace

Myung’s [70] scale, which was revised for the sample of childcare teachers from the previous scale developed for college students and the adult population in Korea [71], was used to measure social support for childcare teachers in the workplace. It consists of 24 questions in total: informational (6 questions), emotional (9 questions), instrumental (4 questions), and appraisal support (5 questions). Examples of questions are as follows: an item for informational support is “they are respectable people with a lot of things to learn”; for emotional support, “they make me feel loved and cared for”; for instrumental support, “they lend me anything I need at any time”; and for appraisal support “they respect me personally.” The original scale was a 5-point Likert scale, but was revised to a 4-point Likert scale for this study, ranging from 1, indicating “strongly disagree”, to 4, indicating “strongly agree.” Cronbach’s α coefficient for the overall social support in the workplace was 0.97 (informational support = 0.92, emotional support = 0.91, instrumental support = 0.79, and appraisal support = 0.86).

#### 2.2.3. Resilience

Resilience was measured with Park’s [72] scale used for childcare teachers in Korea, which was referred to as Ego Resiliency scale (ER) [73]. The items on the ER were drawn from the California Psychological Inventory (CPI) [74] and were translated and adapted for childcare teachers in Korea [72]. The validity and reliability have already been tested in previous studies [72,73,75]. The scale consists of a total of 29 questions, including self-confidence (9 questions), interpersonal relationship efficacy (8 questions), optimism (10 questions), and emotion regulation (2 questions). Each area included the following statements: “I lack self-confidence” for self-confidence; “It is difficult to talk to strangers” for interpersonal relationship efficacy; “I often feel that I have made a mistake in my major selection” for optimism, and “I often get angry” for emotion regulation. The original measure is a 5-point Likert scale, but was revised to a 4-point Likert scale for this study. Cronbach’s α was 0.93 for overall resilience. For the subscales, the Cronbach’s alpha coefficient was 0.85 for self-confidence, 0.85 for interpersonal relationship efficacy, 0.81 for optimism, and 0.60 for emotion regulation, respectively.

### 2.3. Analysis Process

Collected data was analyzed using SPSS 23 and the PROCESS 3.5 macro program developed by Hayes [76]. First, the demographic characteristics of the subjects and descriptive statistics were examined and the internal consistency for each measure was checked by a reliability test using Cronbach’s α coefficient. Second, Pearson’s correlation analysis was conducted to examine the relationship between all variables, while a confirmatory factor analysis was done to validate the distinctiveness of the three variables. Third, the Process macro program was used for the mediation analysis, while the significance of the indirect effect was tested through the bootstrapping method.

## 3. Results

First, results from the descriptive statistics are presented in Table 2. The mean score of social support was 2.94, while among the subscales of social support, the range of the mean score was from 2.88 to 2.97; the mean score of informational support was the highest, while that of instrumental support was the lowest. The mean score of resilience was 2.82 and the range of mean scores of the subscales was from 2.80 to 2.90; the mean score of positive attitudes was the highest, while that of emotion regulation was the lowest. The mean score of self-care was 2.69, and the range of mean scores of the subscales was from 2.37 to 2.99; the mean score of physical activity was the lowest, while that of interpersonal relations was the highest.

Second, Pearson’s correlation analysis was conducted to examine the relationships among childcare teachers’ social support in the workplace, resilience, and self-care (Table 3). The results showed significant and positive correlations between three measures and therefore, Hypothesis 1 was supported.

Next, confirmatory factor analysis (CFA) was performed to see whether the three variables in the model distinctively differentiated from one another (Table 4). The result showed that the fit for the three-factor model was better than that for other models (χ^2^ (63) = 215.55, GFI = 0.94, TLI = 0.96, CFI = 0.97, RMSEA = 0.07).

**Figure 2:**
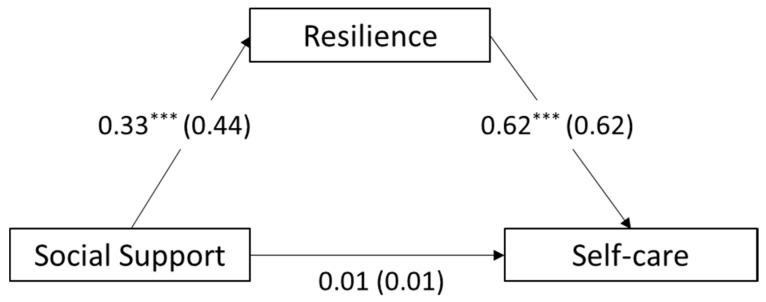
Testing of the Mediation Model. *** *p* < 0.001.

To examine the mediating role of resilience in the path between social support in the workplace and self-care of childcare teachers, the PROCESS 3.5 macro program (Model 4) developed by Hayes [76] was used. The bootstrapping method was used to test the indirect effect of resilience. We tested both models with and without control variables, such as age of childcare teachers and educational attainment, while inserting control variables did not make a significant difference to the results. Hence, the model without control variables, which was simpler, was chosen [77]. Results from the mediation analysis are presented in Table 5 and Figure 2.

Social support for childcare teachers in the workplace had a significant effect on resilience (B = 0.33, β = 0.44, *p* < 0.001; supporting Hypothesis 2a) in the regression model including resilience (M) and social support (X) only. Results from the regression model with all three variables revealed that resilience had a significant effect on self-care (B = 0.62, β = 0.62, *p* <0.001; supporting Hypothesis 2b). Meanwhile, the direct effect of social support was not significant (B = 0.01, β = 0.01, *p* >0.05), which indicated that it was a complete mediation. The bootstrapping result showed that the confidence interval was between 0.14 and 0.27 and confirmed that the mediating role of resilience was significant.

## 4. Discussion

This study investigated the relationship between the self-care of childcare teachers, social support in the workplace, and resilience using a sample of 466 childcare teachers at daycare centers located in Seoul and Gyeonggi Province, South Korea. A discussion on the key findings is as follows. First, the mean score of self-care (m = 2.69) was lower than those reported in previous studies [67,78], with this score indicating that self-care of childcare teachers is inadequate. This may be because childcare teachers in Korea are known to struggle due to a heavy workload, the way they are treated at work, and high levels of stress [79], while the ratio of young children per teacher at daycare centers is higher than in other OECD countries, especially for 3–5 year-olds [21]. As for social support in the workplace, the mean score (m = 2.94) was lower than the recent research (m = 3.73) [64,80]. This may be because the subjects of the study who had higher scores were not residing in Seoul or the Seoul metropolitan area, which is one of the most densely packed cities in the world [81] and thus, the relationship quality among teachers, as well as that between teachers and directors of daycare centers, can be different; it has been reported that urban life in dense cities increases anonymity and stress and eventually, social connections in the community decrease [82]. Furthermore, participants in one of the studies [80] were heavily involved with children with disabilities and thus, fellow teachers might show greater concern and provide more assistance to the participants. The mean score of resilience (m = 2.82) was lower than the score reported in a previous study (m = 3.46) [75]. The result could be due in part to the fact that the subjects in the previous study were still in college; students may show better resilience as an academic environment is less stressful than a workplace and they have no obligation or responsibility for caregiving.

In terms of Hypothesis 1, it was supported by the results showing significant and positive correlations among self-care of childcare teachers, resilience, and social support in the workplace. The findings are in accordance with previous studies of childcare teachers [62,63] as was reviewed in the Introduction. In regard to Hypothesis 2, it was supported as the mediating role of resilience and the significance of the mediation was confirmed. First, a significant and direct effect of social support on the resilience of childcare teachers was found and thus, Hypothesis 2-1 was supported. Results were in line with earlier research [35,64,83] stating that childcare teachers with higher resilience were more likely to perceive the level of social support from fellow childcare teachers in the workplace [83]. Studies have demonstrated that support within organizations had a positive effect on resilience [84] and it was suggested that advice, counseling, praise, and respect from directors and fellow teachers had a positive relationship with childcare teachers’ resilience [61]. Resilience was found to be positively and significantly related to self-care of childcare teachers, which supported Hypothesis 2-2. Studies reported that a higher level of resilience led to better self-care [42,43,57] and the result of the current study was consistent with this.

Resilience showed a complete mediation, while the direct effect of social support in the workplace on self-care was not significant. This may explain the question that previous research addressed as to why colleague assistance programs were not so effective [85,86]. Studies stressed the very low rate of usage of colleague assistance programs among counsellors and therapists; people tended not to seek help even when they were distressed [85]. Furthermore, people had a tendency to ignore the situation and not offer assistance when a colleague seemed emotionally unstable, distressed, or impaired [86,87]. In line with these previous reports, it is expected that childcare teachers, who experienced similar stress from providing caregiving services, may have the same tendency to avoid confronting and discussing their distress with colleagues and subsequently seek help. Therefore, social support from colleagues, fellow teachers, and the director of the daycare center may affect the self-care of childcare teachers only indirectly through resilience, as was found in this study.

### 4.1. Implications

The self-care behavior of professionals who are engaged in a caregiving role was perceived as being selfish as well as a luxury, despite the need for self-care to be considered vital to performing the professional role well and sustaining a state of well-functioning [2]. The result of the study showed a relatively lower level of self-care of childcare teachers compared to other studies using participants working in other occupations (e.g., nurses, students, etc.). Occupations deeply involved with caregiving work such as childcare teachers and therapists have their own unique nature that can be characterized as demanding, stressful, and sometimes even distressing [88]. Childcare teachers may not even be aware of their own needs because the demands of young children come first. Due to either personal predisposition or the training to become caregiving professionals, childcare teachers are likely to choose the needs of children over their own, although they may eventually lose their professional competence as a caregiver if they fail to be aware or ignore those symptoms of distress. In addition, considering that the survey was conducted in Korea, which has had a strong culture of collectivism for a long time [89,90], childcare teachers may feel an even greater difficulty expressing their own distress and seeking personal rest outside work. It should be noted that people are likely to experience distress, burnout, and further impaired professional competence when personal and professional demands and activities are not balanced well in one’s life [2,4]. Thus, education programs focused on self-care for childcare teachers need to be developed, while materials should include content informing childcare teachers that self-care is a crucial part of their professional identities [91,92] and that accepting ongoing self-care behavior is included in the ethics of teachers to provide optimal and effective caregiving to young children. People who participate in the programs may feel less vulnerable in regard to stressful events and their mental health problems [93].

Since social support can be helpful in building and strengthening the resilience of childcare teachers, as was identified in this study, education programs regarding the importance of support from peer groups and directors are urgent in regard to changing the culture of daycare centers (“culture of silence” [2]—not offering assistance and not seeking help, as previously mentioned) and getting rid of blind spots. Childcare teachers may seek help more actively, while colleagues offer help and reach out when noticing symptoms of impairment or distress in fellow teachers. Upon experiencing the benefits of support among colleagues, childcare teachers will be more engaged in supportive relationships at the daycare center and strengthen their ability to bounce back from stressful and challenging adversities, which will thus let them manage their professional life effectively and take care of their personal wellness.

The result of the study, namely discovering the mediating role of resilience in the path between social support and self-care, places greater importance on the resilience of childcare teachers, which was rather recently given attention in Korea compared to other personal traits and characteristics. Teachers with low self-resilience are likely to view their lives negatively, lack self-confidence, be unable to manage or control emotions, and give up easily upon confronting a problem [72]. Childcare teachers with low resilience and poor self-care are likely to become less sensitive and responsive to children’s demands and needs. A decrease in teacher’s sensitivity subsequently leads to negative interactions with young children and results in negative outcomes in children’s socioemotional development [28,29]. Learning skills to improve resilience can boost the ability to cope with stress and maintain emotional stability, as well as physical health [94], which in turn encourages teachers to engage in self-care more actively. Resilience research has been ongoing in the United States, with participants of the resilience program showing increases in self-care (health promotion) behaviors [95]. This program has been applied to various groups and organizations, including schools and colleges, as well as corporations. In Korea, there exist resilience programs for childcare teachers run by government organizations (Childcare Comprehensive Support Centers, Childcare Promotion Centers, and the Ministry of Health and Welfare). However, such programs have only recently been introduced and thus, resilience cannot be improved after participating simply once or twice. Providing ongoing education regarding the importance of resilience, as well as giving opportunities to participate in various programs aimed at building the level of resilience, are required. These programs need to be developed in a way that is tailored to the demands and characteristics of the audience (e.g., differentiated programs in terms of years of work, whether for directors of daycare centers or childcare teachers, for group programs, etc.) and delivered via various ways and media (online vs. offline; mobile phone vs. desktop computers; live stream vs. pre-recorded) to make the programs more easily accessible.

### 4.2. Limitation

The limitations of the study and suggestions for future studies are as follows. First, since this study was conducted on daycare teachers located in Seoul and Gyeonggi Province, there is limited generalizability of the results to other cities and thus, generalization of the results across countries should be done with caution. Second, the measure for self-care used in this study was not the one developed specifically for childcare teachers. Generating and developing a scale for childcare teachers would give greater insight since self-care encompasses various domains of life [4]. Hence, a scale for childcare teachers, such as the one developed for psychologists [96], may allow more accurate assessment and capture detailed information, especially in the self-care behaviors related to professional life. Third, this study included social support in the workplace only. Support from family and friends was not considered, although support from close relationships may have an even greater impact on self-care and resilience [42]. In addition, attempts to explore more various predictors of self-care of childcare teachers, as well as the underlying mechanism that can strengthen the level of self-care, need to be continued. Along with the recent movement of policy promoting the rights of childcare teachers, testing other important variables that can influence self-care, including macro systems such as government policy and regulations from the perspective of Bronfenbrenner’s [97] ecological system approach, is necessary. Fourth, in this study, more than 80% of teachers were married, while a large number of them played the role of caregiver for their children at home. Marriage doubles the burden of care and thus, it would be meaningful to focus on married childcare teachers’ self-care in a follow-up study. Lastly, all subjects in this study were female childcare teachers. Although the number of male teachers currently accounts for less than 2% in Korea [98], it would be desirable to explore self-care of male teachers considering the growing percentage of male teachers in recent years, as well as gender differences in the key variables used in this study. It was reported that men showed greater social support [99,100]. The level of self-care or self-management in daily life was reported to be higher for females [101,102]. As for resilience, studies have shown mixed results; some reported higher levels of resilience for males [100,103], while others showed little difference in regard to gender [104]. Therefore, male childcare teachers may show different results.

## 5. Conclusions

This study discovered that resilience of childcare teachers was a pathway by which social support in workplace influenced teachers’ self-care. Little has been known regarding the relationship between self-care, resilience, and social support in the workplace, in particular for a childcare teacher, which is one of the most stressful occupations [52,79]. This study contributed to advancing the understanding of underlying mechanisms that can enhance self-care of childcare teachers. The results of the study suggest that social support in the workplace, in other words, structural support, is required for teachers to enhance their levels of resilience. As such, resilient childcare teachers can carry out adequate self-care, which leads them to personal wellbeing, as well as more effective professional functioning. This will eventually help lower the costs of healthcare from a societal perspective.

## Figures and Tables

**Table 1:** Characteristics of the Participants.

Variable	Category	Frequency (%)
Age	20–29 years old 30–39 years old 40 years old and above	78 (16.7) 129 (27.7) 259 (55.6)
Work experience	Less than 5 years 5–10 years More than 10 years	209 (44.8) 156 (33.5) 101 (21.7)
Daycare center type	Public Private In-home	88 (18.9) 193 (41.4) 185 (39.7)
Marital status	Married Single	375 (80.5) 91 (19.5)
Educational attainment	Less than high school 2-year college 4-year university bachelor’s degree Graduate school or higher	42 (9.0) 214 (45.9) 193 (41.4) 17 (3.6)
Monthly income (Korean won; KRW)	1,000,000–2,000,000 (approximately US$900–1800) 2,000,000–3,000,000 (approximately US$1800–2700) More than 3,000.000 (approximately US$2700)	261 (56.0) 189 (40.6) 16 (3.4)
Working hours per day	Less than 9 hours 9 hours More than 9 hours	95 (20.4) 331 (71.0) 40 (8.6)
Total		466 (100)

**Table 2:** Descriptive Statistics.

Variable	Minimum	Maximum	M	SD	Skewness	Kurtosis
Social support	1.00	4.00	2.94	0.43	−0.27	1.97
Resilience	1.81	3.85	2.82	0.32	0.49	1.15
Self-care	1.79	4.00	2.69	0.32	0.65	1.96

**Table 3:** Correlations among the Variables.

Variable	Social Support	Resilience	Self-Care
Social support	-		
Resilience	0.44 ***	-	
Self-care	0.29 ***	0.63 ***	-

*** *p* < 0.001.

**Table 4:** Results of the Confirmatory Factor Analysis.

Number of Factors	X^2^	df	GFI	TLI	CFI	RMSEA
1-factor	1561.71	66	0.63	0.70	0.70	0.22
2-factor	897.11	65	0.79	0.76	0.83	0.17
3-factor	215.55	63	0.94	0.96	0.97	0.07

Note: GFI = Goodness-of-Fit Index; TLI = Tucker-Lewis Index; CFI = Comparative Fit Index; RMSEA = Root Mean Square Error of Approximation.

**Table 5:** Testing of the Mediation Model (*N* = 466).

Antecedent	Consequent							
	Resilience (M)			Self-Care (Y)				
	B (β)	SE	*p*	B (β)		SE		*p*
Social support (X)	0.33 *** (0.44)	0.03	<0.001	0.01 (0.01)		0.03		0.74
Resilience (M)	-	-	-	0.62 *** (0.62)		0.04		<0.001
Constant	1.85 ***	0.09	<0.001	0.93 ***		0.11		<0.001
Model Summary	R^2^ = 0.19 F(1, 464) = 110.00, *p* < 0.001			R^2^ = 0.40 F(2, 463) = 151.36, *p* < 0.001				
Significance test of the indirect effect (bootstrap samples = 5000; level of confidence 95%)								
Effect size (standardized size)			BootSE		BootLLCI		BootULCI	
0.20 (0.27)			0.03		0.14		0.27	

Note: *** *p* < 0.001. ‘SE’ denotes standard errors. “LLCI” denotes “Lower limit confidence interval,” and “ULCI” denotes “Upper limit confidence interval.”
